# Prevalence, Clinical Characteristics, and Predictors of Difficult-to-Treat Inflammatory Bowel Disease in a Real-World Taiwanese Cohort

**DOI:** 10.3390/life16020197

**Published:** 2026-01-24

**Authors:** Shun-Wen Hsiao, Pei-Yuan Su, Chen-Ta Yang, Yang-Yuan Chen, Hsu-Heng Yen

**Affiliations:** 1 Division of Gastroenterology, Department of Internal Medicine, Changhua Christian Hospital, Changhua 500, Taiwan; 2 Department of Post-Baccalaureate Medicine, College of Medicine, National Chung Hsing University, Taichung 402, Taiwan; 3 Artificial Intelligence Development Center, Changhua Christian Hospital, Changhua 500, Taiwan

**Keywords:** Crohn’s disease, ulcerative colitis, inflammatory bowel disease, difficult to treat

## Abstract

A subset of patients with inflammatory bowel disease (IBD) remains refractory to treatment despite multiple lines of advanced therapies. These patients are often categorized as having difficult-to-treat (DTT) IBD. We retrospectively analyzed 354 patients with IBD (including 112 with Crohn’s disease [CD] and 242 with ulcerative colitis [UC]) from a real-world cohort. Baseline demographic and disease characteristics, treatment history, and outcomes were compared between the DTT-IBD and non-DTT-IBD groups. Logistic regression analysis was performed to identify factors associated with DTT-IBD in CD and UC cohorts. Approximately 10.6% of the patients exposed to advanced therapy fulfilled the definition of DTT-IBD (CD: 9.8%, UC: 11.4%). Compared with patients with non-DTT-IBD, those with DTT-IBD exhibited a significantly higher exposure to multiple biologic classes, including antitumor necrosis factor (94.1% vs. 59.0%), anti-integrin (94.1% vs. 47.2%), anti-interleukin-12/23 (88.2% vs. 19.4%), and Janus kinase inhibitors (35.3% vs. 0.7%). The DTT-IBD group had a significantly lower clinical remission rate at the last follow-up than the non-DTT-IBD group (52.9% vs. 85.4%, *p* = 0.001). A longer interval from diagnosis to the initiation of advanced therapy was independently associated with DTT-IBD in CD (OR: 1.014 per month, 95% CI: 1.001–1.026, *p* = 0.026). No significant predictors for UC were identified. In conclusion, DTT-IBD, characterized by extensive biologic exposure and suboptimal long-term remission rates, accounts for approximately 10% of patients with IBD receiving advanced therapy. In CD, delayed initiation of advanced therapy may contribute to refractoriness. These findings emphasize the unmet need for earlier therapeutic intervention, better predictive markers of treatment response, and novel therapeutic mechanisms.

## 1. Introduction

Inflammatory bowel disease (IBD), encompassing Crohn’s disease (CD) and ulcerative colitis (UC), is a chronic, immune-mediated disorder characterized by relapsing intestinal inflammation [1,2,3]. Over the last two decades, the therapeutic landscape of IBD has transformed with the advancements of biologic agents and small-molecule therapy targeting diverse immune pathways, including antitumor necrosis factor (TNF), anti-integrin, anti-interleukin (IL)-12/23, anti-IL-23, and Janus kinase (JAK) inhibitors [4,5,6]. These treatments have significantly improved the rates of remission and reduced the need for surgery in these patients [7]. Despite the rapid diversification of the therapeutic armamentarium, both clinical trial data and longitudinal real-world evidence consistently highlight a persistent “therapeutic ceiling” in IBD management. Even with the emergence of novel agents targeting diverse molecular pathways, primary non-response and secondary loss of response remain prevalent challenges. Clinical remission rates in pivotal phase III trials frequently plateau between 40% and 60%, suggesting that current monotherapies may only modulate a fraction of the complex, redundant inflammatory cascades that drive disease pathogenesis. Consequently, a substantial subset of patients remains refractory to treatment despite sequential exposure to multiple classes of advanced therapies. This cohort—increasingly recognized as “difficult-to-treat” (DTT)—often experiences a cycle of “revolving door” biologic switching, where the exhaustion of available mechanisms of action leads to diminished clinical returns and an increased risk of disease complications. The persistence of this refractory population underscores a critical gap in current treatment strategies and necessitates a deeper investigation into the predictors of multi-drug failure [8].

The international expert groups have proposed the concept of difficult-to-treat (DTT) IBD to characterize this complex population. Patients with DTT-IBD are not usually successfully managed with multiple biologics or small-molecule therapy. Further, they experience persistent disease activity and require repeated treatment escalations or surgical interventions [9]. The recognition of DTT-IBD is clinically important, as it identifies patients at high risk for poor long-term outcomes, including hospitalization, surgery, and impaired quality of life [10,11]. Nevertheless, data on the prevalence, clinical characteristics, and predictors of DTT-IBD remain limited, particularly in Asian populations whose disease phenotypes and treatment patterns may differ from those of Western cohorts [12,13,14,15]. Compared with Western cohorts, Asian patients with IBD have been reported to exhibit distinct disease phenotypes, including lower rates of penetrating and perianal Crohn’s disease, different distributions of UC extent, and unique genetic and immunologic backgrounds [14]. In addition, treatment strategies in Asia are often influenced by delayed biologic initiation and stricter reimbursement policies, which may substantially alter therapeutic sequencing and the evolution toward treatment refractoriness [15].

Previous studies have revealed that early initiation of effective therapy may alter the natural history of IBD, particularly in CD, where diagnostic delay is associated with complications such as structuring, fistulization, and bowel resection [16]. However, the extent to which delayed treatment contributes to the development of DTT-IBD has not been systematically evaluated. Moreover, although biologic sequencing is common in clinical practice, the cumulative impact of multiple drug exposures on subsequent treatment refractoriness and long-term remission rates is not completely understood [17,18].

The current study aimed to investigate the prevalence, clinical features, and potential predictors of DTT-IBD resulting from treatment failures in a real-world cohort of patients from Taiwan with CD and UC who were receiving advanced therapy.

By identifying the risk factors for treatment refractoriness, particularly those related to treatment timing and cumulative drug exposure, our findings can provide insights that may inform earlier intervention strategies and guide the development of more effective therapeutic approaches for this difficult-to-manage patient population.

## 2. Materials and Methods

### 2.1. Study Design and Population

This retrospective cohort study was conducted at Changhua Christian Hospital. Consecutive patients diagnosed with IBD, including those with CD and UC, between January 2010 and December 2024 were examined. In total, 354 patients were included in the final analysis (CD, n = 112; UC, n = 242). Patients with a confirmed diagnosis of IBD based on standard clinical, endoscopic, radiologic, and histopathologic criteria were eligible for this study. Follow-up continued until December 2024, loss to follow-up, or death, whichever occurred first.

### 2.2. Data Collection

Demographic and clinical variables, including age, sex, disease duration, time interval from symptom onset to diagnosis, disease location and phenotype, history of bowel resection, and presence of perianal disease, were extracted from the electronic medical records. The treatment-related variables included the use of advanced therapies (anti-TNF, anti-integrin, anti-IL-12/23, anti-IL-23, and JAK inhibitors), number and sequence of advanced therapies, and time interval from diagnosis to the initiation of advanced therapy. Clinical remission status at the last follow-up was also recorded.

### 2.3. Definition of Difficult-to-Treat-IBD

DTT-IBD was defined using the international consensus criteria, encompassing patients with (a) persistent active disease despite exposure to at least two advanced therapies with different mechanisms of action or (b) postoperative recurrence of Crohn’s disease after two or more intestinal resections [9]. Patients with CD and UC were evaluated separately for the presence of DTT-IBD.

### 2.4. Outcomes

The primary outcome was the prevalence of DTT-IBD among patients treated with advanced therapies. The secondary outcomes were the demographic and clinical characteristics of the DTT-IBD and non-DTT-IBD patients, treatment patterns, and clinical remission at the last follow-up. Logistic regression analyses were performed to identify the independent predictors of DTT-IBD in CD and UC.

### 2.5. Statistical Analysis

Continuous variables were presented as medians with interquartile ranges (IQRs) and compared using the Mann–Whitney U test. Categorical variables were expressed as frequencies and percentages and compared using the chi-square test or Fisher’s exact test, as appropriate. To identify the potential predictors of DTT-IBD, logistic regression analyses were conducted separately for CD and UC, with results expressed as odds ratios (ORs) with 95% confidence intervals (CIs). Variables with a *p*-value of <0.10 in the univariate analyses were entered into the multivariate models. A two-sided *p*-value of <0.05 indicated statistically significant differences. Statistical analyses were performed using SAS version 9.4 (SAS Institute Inc., Cary, NC, USA).

## 3. Results

### 3.1. Clinical Features of the Population with IBD

As shown in Table 1, 354 patients, including 112 with CD (31.6%) and 242 with UC (68.4%), were analyzed. The median age at diagnosis was similar between patients with CD and those with UC (37.3 [IQR: 27.7–53.1] vs. 40.0 [IQR: 30.8–50.5] years, *p* = 0.63). However, patients with CD (median: 5.5 months, IQR: 2.0–33.5) had a significantly longer time interval from symptom onset to diagnosis than those with UC (median: 1.0 months, IQR: 1.0–6.0, *p* < 0.001). Patients with CD also had significantly higher rates of complications, including bowel resection (35.7% vs. 1.2%, *p* < 0.001) and perianal disease (14.3% vs. 0%, *p* < 0.001).

**Figure 1:**
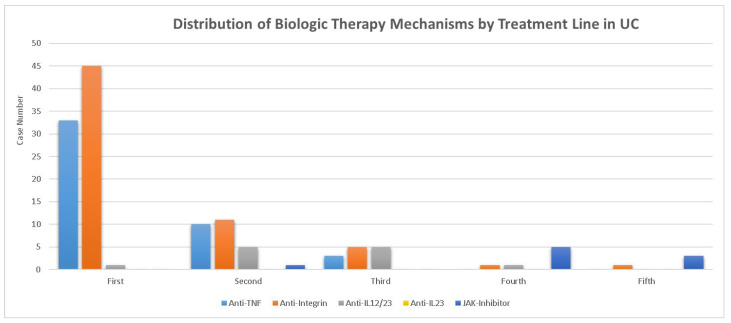
Distribution of biologic therapy mechanisms by treatment line in ulcerative colitis. Number of patients receiving each biologic or small-molecule therapy (anti-TNF, anti-Integrin, anti-IL-12/23, anti-IL-23, and JAK inhibitor) across the first- to fifth-line treatments in ulcerative colitis (UC).

**Figure 2:**
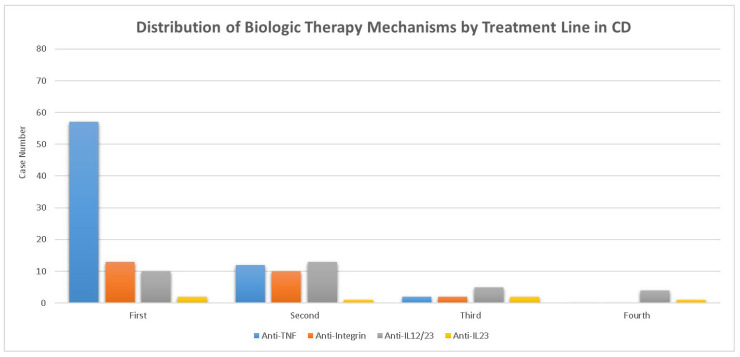
Distribution of biologic therapy mechanisms by treatment line in Crohn’s disease. Number of patients receiving each biologic or small-molecule therapy (anti-TNF, anti-integrin, anti-IL-12/23, anti-IL-23, and JAK inhibitor) across the first- to fourth line treatments in Crohn’s disease.

Regarding advanced therapy, 82 patients with CD (73.2%) and 79 with UC (32.6%) received at least one biologic or small-molecule therapy. Figure 1 depicts the distribution of the mechanism of biologic therapy in UC by treatment line. In contrast, patients with UC were more commonly treated with anti-integrin agents (76.7%) and less frequently with anti-IL-12/23 (15.2%) or JAK inhibitors (9.7%). The distribution of treatment lines (first to fifth lines) did not differ significantly between patients with CD and those with UC. Figure 2 shows the distribution of the mechanism of biologic therapy in CD by treatment line. In patients with CD, anti-TNF was the most frequently used agent (73.2%), followed by anti-IL-12/23 (37.8%) and anti-integrin (30.5%).

### 3.2. Clinical Features and Predictors of Difficult-to-Treat-IBD

As shown in Table 2, the prevalence of DTT-IBD among patients who were exposed to advanced therapies was 10.6%. This corresponds to rates of 9.8% in CD and 11.4% in UC, with no significant difference observed between the two disease types (*p* = 0.74). In our cohort, no patients met the DTT-IBD criterion of postoperative recurrence after intestinal resection; therefore, DTT-IBD classification was based exclusively on treatment refractoriness to advanced therapies. Patients with DTT-IBD and those with non-DTT-IBD did not significantly differ in terms of sex distribution, age at diagnosis, and disease phenotype or extent. Compared with patients with non-DTT-IDB, those with DTT-IDB had significantly higher rates of exposure to anti-TNF (94.1% vs. 59.0%, *p* = 0.005), anti-integrin (94.1% vs. 47.2%, *p* < 0.001), anti-IL-12/23 (88.2% vs. 19.4%, *p* < 0.001), anti-IL-23 (17.6% vs. 2.1%, *p* = 0.001), and JAK inhibitors (35.3% vs. 0.7%, *p* < 0.001). At the last follow-up, clinical remission was significantly less common in patients with DTT-IDB than in those with non-DTT-IDB (52.9% vs. 85.4%, *p* = 0.001).

### 3.3. Predictors of Difficult-to-Treat IBD

In the logistic regression analyses stratified by disease type, as shown in Table 3, delayed initiation of advanced therapy was independently associated with DTT-IBD in CD (OR: 1.014 per month, 95% CI: 1.001–1.026, *p* = 0.026). Neither symptom-to-diagnosis interval nor the choice of first-line advanced therapy (anti-TNF vs. anti-integrin) was associated with treatment refractoriness in CD. Conversely, among patients with UC, no significant predictors of DTT-IBD, including diagnostic delay, disease extent, and type of first-line therapy, were identified.

## 4. Discussion

In this real-world Taiwanese cohort, approximately 10% of patients with IBD who were exposed to advanced therapy met the criteria for DTT-IBD. This prevalence is comparable with that in previous reports from Western cohorts [12], which indicated that DTT-IBD represents a clinically significant and consistent subset across different geographic and ethnic groups. Importantly, our findings emphasize that patients with DTT-IBD had been exposed to multiple biologic mechanisms. However, their rates of clinical remission remained substantially lower than those observed in patients with non-DTT-IBD. This finding underscores the unmet therapeutic need in this population.

The identification of DTT-IBD as a distinct clinical entity has refined the characterization of refractory disease beyond simple treatment failure. To date, international consensus defines DTT-IBD as persistent disease activity despite exposure to two or more advanced therapies with different mechanisms of action. The prevalence (approximately 10%) observed in our cohort aligns with estimates from Latin America (13%) [13] and Italian tertiary centers (24.8%) [12], suggesting that difficult-to-treat IBD is not confined to specific healthcare systems or populations. Differences in prevalence likely reflect heterogeneity in therapeutic access, reimbursement policies, treatment sequencing strategies, and clinical practice patterns across health systems, rather than purely disease biology.

However, our findings extend this concept to an Asian population, where patterns of disease phenotype, therapeutic access, and biologic sequencing are influenced by distinct genetic backgrounds and national reimbursement policies. In Taiwan, reimbursement for the use of biologics remains tightly regulated, often resulting in delayed initiation of advanced therapy. This real-world delay may contribute substantially to the refractory disease burden observed [19,20].

A key finding of our analysis is that a longer interval from diagnosis to the initiation of advanced therapy was independently associated with the development of DTT-IBD in patients with CD. This finding supports the window of opportunity hypothesis, which suggests that early intervention during the inflammatory phase may modify the disease trajectory before the emergence of irreversible fibrostenotic or penetrating complications [21]. Similar associations have been observed in previous studies, which showed that treatment delay is associated with irreversible bowel damage, higher rates of surgery, and poorer therapeutic response [22,23,24,25,26]. Our data reinforce the importance of early treatment escalation, particularly in patients with CD. This finding indicates that chronic uncontrolled inflammation fosters cumulative structural and immunologic remodeling that limits therapeutic reversibility. These findings have important clinical and policy implications. Considering that the National Health Insurance of Taiwan requires stringent criteria for biologic reimbursement, with the need for 6 months of conventional therapy before initiating advanced therapy, the prolonged approval process may inadvertently defer optimal therapy. Reducing administrative barriers and promoting early escalation for high-risk phenotypes—such as young age at onset, ileocolonic disease, and penetrating behavior—could mitigate progression to refractory disease. Moreover, the association between delayed therapy and DTT-IBD emphasizes the need for systematic early referral pathways and risk-stratified management algorithms in Asian healthcare settings.

In contrast, there were no significant predictors of DTT-IBD among patients with UC. This discrepancy may reflect fundamental differences in the disease biology between UC and CD and the need for various treatment strategies across the two conditions [27,28]. Recent multi-omics studies have revealed that treatment refractoriness in UC is driven less by conventional clinical factors and more by molecular endotypes that involve mitochondrial dysfunction, epithelial barrier defects, and altered microbial composition [29,30,31]. The lack of identifiable predictors supports this concept, implying that traditional clinical variables—such as disease extent or diagnostic delay—are insufficient to capture UC refractoriness. Future research incorporating mucosal transcriptomics, microbiome profiling, and immunophenotyping should be performed to delineate the molecular subtypes predictive of DTT behavior.

Another salient observation is the extensive therapeutic exposure among patients with DTT-IBD, many of whom had cycled via anti-TNF, anti-integrin, anti-IL-12/23, anti-IL-23, and JAK inhibitors. Despite this aggressive treatment, >40% of patients did not achieve remission at the last follow-up. These findings highlight the diminishing therapeutic durability of successive biologic classes and the concept of mechanistic exhaustion, wherein the modulation of the cumulative immune pathway may lead to partial resistance across drug targets. This raises the question of whether sequencing or combination strategies can improve outcomes in the future [32,33,34]. Emerging therapies such as selective JAK inhibitors, S1P receptor modulators, and bispecific antibodies may provide new avenues. However, evidence in the population with DTT-IBD remains limited [35].

This study has several clinical implications. First, our data reinforce the need for early, risk-stratified intervention in CD and to consider early treatment escalation in patients at risk of poor outcomes [36,37]. Multidisciplinary assessment—including gastroenterologists, surgeons, nutritionists, and psychologists—should be advocated for this DTT-IBD management. Second, the management of DTT-IBD will likely require a precision medicine approach, integrating clinical, genetic, and microbiome-derived biomarkers to guide therapy selection [38,39,40,41,42,43]. Artificial intelligence-based predictive models in the future, which can synthesize longitudinal clinical and omics data, may further personalize treatment selection and predict loss of response. Third, at the policy level, health authorities should consider that delayed biologic access may contribute to irreversible disease progression and higher cumulative costs. Facilitating earlier initiation of biologic treatment for high-risk patients with CD could ultimately reduce surgical and hospitalization expenditures, aligning clinical benefit with health-economic rationale [44,45].

The present study faced a number of limitations that should be recognized. First, this study’s retrospective, single-center design limited the generalizability of the results. This reflects the real-world prevalence of this condition rather than a selection bias. Second, the relatively small number of DTT-IBD cases reduced statistical power, precluding the identification of weaker predictors or phenotype-based predictors. Parigi et al. [12] analyzed a substantially larger Western cohort of 1736 IBD patients and identified several UC- and CD-specific risk phenotypes associated with DTT-IBD, including left-sided and extended colitis in UC and stricturing, penetrating, and ileocolonic/upper gastrointestinal involvement and perianal disease in CD. These findings underscore the need for larger, multicenter cohorts to delineate phenotype-based predictors and therapeutic trajectories in DTT-IBD within Taiwan. Third, this study is retrospective; endoscopic and biomarker evaluations were not uniformly performed in routine care. Therefore, disease activity and remission were assessed primarily based on documented clinical assessments in the medical record.

Fourth, despite adopting the international consensus definition of DTT-IBD, differences in physician practice and national reimbursement criteria may influence classification. For example, the National Health Insurance of Taiwan does not permit dose escalation or interval shortening for biologics, which may accelerate therapeutic exhaustion, thereby predisposing patients to DTT classification despite suboptimal exposure. Nonetheless, these data can provide valuable real-world evidence from an underrepresented Asian cohort and can be a foundation for prospective multicenter studies. Nevertheless, future research should incorporate objective endpoints, longitudinal follow-up, and integrative multi-omics analyses to elucidate the mechanistic determinants of refractory disease and to validate predictive models for early identification.

## 5. Conclusions

DTT-IBD, characterized by extensive biologic exposure and poor remission rates, affects approximately 10% of patients with IBD receiving advanced therapy in Taiwan. Delayed initiation of advanced therapy was an independent predictor of refractoriness in CD, emphasizing the clinical and policy importance of early therapeutic escalation. Addressing the challenges associated with DTT-IBD will require not only novel mechanisms of action but also a precision medicine framework integrating molecular biomarkers, real-world analytics, and equitable access to timely treatment. Collectively, these strategies may be promising for transforming DTT-IBD from a refractory end state into a preventable outcome.

## Figures and Tables

**Table 1:** Baseline Characteristics of the Population with IBD.

Variables	Patients with CD (n = 112, 31.64%)	Patients with UC (n = 242, 68.36%)	*p*-Value
Male sex, n (%)	80 (71.4%)	148 (61.2%)	0.061
Age at diagnosis, year, median (IQR)	37.29 (27.67–53.05)	40.00 (30.83–50.50)	0.634
Time interval from the onset of IBD symptoms to diagnosis, month, median (IQR)	5.50 (2.00–33.50)	1 (1.00–6.00)	<0.001
UC extent, E1/E2/E3, n (%)		44 (18.2%)/82 (33.9%)/116 (47.9%)	
CD phenotype, B1/B2/B3, n (%)	40 (35.7%)/36 (32.1%)/ /36 (32.1%)		
CD location, L1/L2/L3/L4, n (%)	37 (33.0%)/11 (9.8%)/ 57 (50.9%)/7 (6.2%)		
History of bowel resection, n (%)	40 (35.7%)	3 (1.2%)	<0.001
Perianal disease, n (%)	16 (14.3%)	0 (0%)	<0.001
Use of advanced therapy, n (%)	82 (73.21%)	79 (32.64%)	<0.001
Anti-TNF, n (%) *	60 (73.17%)	41 (51.90)	0.005
Anti-integrin, n (%) *	25 (30.49%)	59 (76.68%)	<0.001
Anti-IL-12/23, n (%) *	31 (37.80)	12 (15.19%)	0.001
Anti-IL-23, n (%) *	6 (7.32%)	0 (0%)	0.015
JAK inhibitors, n (%) *	0 (0%)	7 (9.72%)	0.006
Mechanisms of advanced therapy used			0.441
1	51 (62.2%)	52 (65.8%)	
2	23 (28.0%)	18 (22.8%)	
3	7 (8.5%)	5 (6.3%)	
4	1 (1.2%)	4 (5.1%)	
Difficult-to-treat IBD	8/82 (9.78%)	9/79 (11.40%)	0.736

Abbreviations: IBD, inflammatory bowel disease; CD, Crohn’s disease; UC, ulcerative colitis; TNF, tumor necrosis factor; IL, interleukin; JAK, Janus kinase. * The population parameter was user of advanced therapy. UC extent (Montreal classification): E1, ulcerative proctitis; E2, left-sided colitis (distal to the splenic flexure); and E3, extensive colitis (proximal to the splenic flexure). CD phenotype (Montreal classification): B1, non-stricturing, non-penetrating; B2, stricturing; and B3, penetrating disease. CD location (Montreal classification): L1, ileal; L2, colonic; L3, ileocolonic; and L4, upper gastrointestinal involvement.

**Table 2:** Difficult-to-treat IBD vs. non-difficult-to-treat IBD.

Variables	DTT-IBD (n-17)	Non-DTT IBD (n = 144)	*p*-Value
**Demographics**			
Crohn’s disease, n (%)	8 (47.1%)	9 (52.9%)	0.736
Male sex, n (%)	11 (64.7%)	99 (68.7%)	0.735
Age at diagnosis, y, median (IQR)	34.17 (19.81–45.44)	36.30 (27.29–47.91)	0.180
**Disease History**			
Symptom onset to diagnosis, mo, median (IQR)	1.00 (1.00–19.00)	2.50 (1.00–15.00)	0.765
Diagnosis to advanced therapy, mo, median (IQR)	61.00 (18.25–141.50)	16.50 (3.00–75.00)	0.182
UC extent, E1/E2/E3, n (%)	0 (0%)/4 (15.4%)/5 (55.6%)	6 (8.5%)/22 (31.0%)/43 (60.6%)	0.538
CD phenotype, B1/B2/B3, n (%)	1 (12.5%)/3 (37.5%) /4 (50%)	18 (24.3%)/27 (36.5%)/ /29 (39.2%)	0.724
CD location, L1/L2/L3/L4, n (%)	3 (37.5%)/0 (0%)/ 5 (62.5%)/0 (0%)	23 (31.1%)/7 (9.5%)/ 37 (50.0%)/7 (9.5%)	0.609
History of bowel resection, n (%)	4 (23.5%)	35 (24.3%)	0.944
Perianal disease, n (%)	1 (5.9%)	14 (9.7%)	0.608
Late biologic therapy (>12 months), n (%)	14 (82.4%)	79 (54.9%)	0.031
**Advanced Therapy Exposure, n (%)**			
Anti-TNF, n (%)	16 (94.1%)	85 (59.0%)	0.005
Anti-integrin, n (%)	16 (94.1%)	68 (47.2%)	<0.001
Anti-IL-12/23, n (%)	15 (88.2%)	28 (19.4%)	<0.001
Anti-IL-23, n (%)	3 (17.6%)	3 (2.1%)	0.001
JAK inhibitors, n (%)	6 (35.3%)	1 (0.7%)	<0.001
**Clinical remission at the last follow-up, n (%)**	9 (52.9%)	123 (85.4%)	0.001

Abbreviations: IBD, inflammatory bowel disease; CD, Crohn’s disease; UC, ulcerative colitis; DTT, difficult-to-treat; TNF, tumor necrosis factor; IL, interleukin; JAK, Janus kinase.

**Table 3:** Logistic regression analysis of DTT-IBD in patients with CD and UC.

Predictor	Odds Ratio (95% CI)	*p*-Value
**Crohn’s Disease**		
Age at diagnosis (year)	0.991 (0.948–1.035)	0.682
Female sex	0.900 (0.168–4.832)	0.902
CD behavior: B1 vs. B2/B3	0.444 (0.051–3.860)	0.462
Perianal disease	0.612 (0.070–5.387)	0.658
Time from symptom onset to diagnosis (per month)	1.000 (0.983–1.016)	0.953
Time from diagnosis to advanced therapy (per month)	1.014 (1.001–1.026)	0.026
First-line agent: anti-integrin vs. others	0.738(0.083–6.560)	0.785
**Ulcerative Colitis**		
Age at diagnosis (year)	0.946 (0.886–1.010)	0.097
Female sex	1.440 (0.354–5.856)	0.610
Disease extent: E1/2 vs. E3	1.200 (0.296–4.862)	0.798
Time from symptom onset to diagnosis (per month)	0.990 (0.944–1.037)	0.667
Time from diagnosis to advanced therapy (per month)	1.003 (09996–1.011)	0.411
First-line biologic: anti-integrin vs. others	1.767 (0.437–7.153)	0.425

## Data Availability

The data that support the findings of this study are available from the corresponding author upon reasonable request.

## Abbreviations

The following abbreviations are used in this manuscript:

CD	Crohn’s disease
UC	Ulcerative colitis
IBD	Inflammatory bowel disease
DTT	Difficult to treat
